# Implementing a sustainable health insurance system in Cambodia: a study protocol for developing and validating an efficient household income-level assessment model for equitable premium collection

**DOI:** 10.1186/s12939-020-1126-8

**Published:** 2020-01-31

**Authors:** Haruyo Nakamura, Floriano Amimo, Siyan Yi, Sovannary Tuot, Tomoya Yoshida, Makoto Tobe, Shuhei Nomura

**Affiliations:** 1 Department of Global Health Policy, Graduate School of Medicine, The University of Tokyo, 7-3-1 Hongo, Bunkyo-ku, Tokyo, 113-0033 Japan; 2 Faculty of Medicine, Eduardo Mondlane University, Maputo, Mozambique; 3 KHANA Center for Population Health Research, Phnom Penh, Cambodia; 4 Center for Global Health Research, Touro University California, Vallejo, CA USA; 5 Saw Swee Hock School of Public Health, National University of Singapore and National University Health System, Singapore, Singapore; 6 Japan International Cooperation Agency, Tokyo, Japan; 7 Research Center for Health Policy and Economics, Hitotsubashi Institute for Advanced Study, Hitotsubashi University, Tokyo, Japan; 8 Department of Health Policy and Management, School of Medicine, Keio University, Tokyo, Japan

**Keywords:** Health financing, Financial sustainability, Health insurance, Contribution, Income-level assessment, Equity, Cambodia

## Abstract

**Background:**

As elsewhere in low- and middle-income countries, due to limited fiscal resources, universal health coverage (UHC) remains a challenge in Cambodia. Since 2016, the National Social Security Fund (NSSF) has implemented a social health insurance scheme with a contributory approach for formal sector workers. However, informal sector workers and dependents of formal sector workers are still not covered by this insurance because it is difficult to set an optimal amount of contribution for such individuals as their income levels are inestimable. The present study aims to develop and validate an efficient household income-level assessment model for Cambodia. We aim to help the country implement a financially sustainable social health insurance system in which the insured can pay contributions according to their ability.

**Methods:**

This study will use nationally representative data collected by the Cambodia Socio-Economic Survey (CSES), covering the period from 2009 to 2019, and involving a total of 50,016 households. We will employ elastic net regression analysis, with per capita disposable income based on purchasing power parity as the dependent variable, and individual and community-level socioeconomic and demographic characteristics as independent variables. These analyses aim to create efficient income-level assessment models for health insurance contribution estimation. To fully capture socioeconomic heterogeneity, sub-group analyses will be conducted to develop separate income-level assessment models for urban and rural areas, as well as for each province.

**Discussion:**

This research will help Cambodia implement a sustainable social health insurance system by collecting optimal amount of contributions from each socioeconomic group of the society. Incorporation of this approach into existing NSSF schemes will enhance the country’s current efforts to prevent impoverishing health expenditure and to achieve UHC.

## Background

Globally, approximately 180 million individuals are estimated to be facing catastrophic health expenditure each year [1]. Therefore, at the General Assembly held in December 2012, the United Nations set the achievement of universal health coverage (UHC) as a new common agenda for the global community [2]. Nevertheless, for low- and middle-income countries (LMICs), it is financially challenging to establish a publicly funded system to achieve UHC targets due to insufficient tax revenue. Accordingly, some LMICs have chosen to adopt a contributory health insurance system to effectively mobilize domestic financing resources [3].

A contributory health insurance is typically introduced for the population employed in the formal sector, such as civil servants and private sector workers, since insurance contributions can be deducted from their salaries. Meanwhile, most LMICs simultaneously provide social and health assistance to poor individuals, often in collaboration with development partners. As a result, non-poor informal sector workers and dependents of formal sector workers are left uninsured [3]. Countries that adopt a contributory health insurance system to cover the general population usually collect a fixed amount of contribution, which is set at a level that the lowest-income group can afford. Such a practice, however, endangers the financial sustainability of the insurance fund or imposes heavy burden of subsidy on the government, whose fiscal resources are already limited [3]. Therefore, there is an urgent need to develop a mechanism to assess household income level to collect contributions according to one’s ability to pay, while ensuring financial sustainability of the insurance fund and placing minimum burden on the governments of LMICs.

Since 2016, in Cambodia, a lower-middle-income country [4], the National Social Security Fund (NSSF) has provided health insurance coverage to formal sector workers. In contrast, poor households are covered by the Health Equity Fund (HEF), the co-financing mechanism of the government and development partners. The government plans to extend the NSSF health insurance system to the rest of the population by 2025 [5]. In December 2017, approximately 31% of Cambodian citizens were covered either by the NSSF health insurance or HEF [6]. Thus, nearly 70% of the population is yet to be covered by health insurance.

The National Institute of Statistics (NIS) under the Ministry of Planning of Cambodia conducts the Cambodia Socio-Economic Survey (CSES) each year, to specifically estimate household income in the country, the key information required for equitable contribution estimation [7]. However, this survey is composed of lengthy questionnaires, which require an average of 2 days to complete in each household. Therefore, the CSES questionnaires are unlikely to be utilized regularly by local administrative staff to estimate health insurance contribution. A more efficient tool with limited number of indices to allow an easy, quick, but accurate assessment of the household income level is therefore needed in Cambodia. By identifying the income level of households efficiently, the emerging insurance model could allow the redistribution of wealth because larger contributions could be collected from individuals with higher income as compared to that collected from those with lower income, while simultaneously ensuring equity in access to health care. Studies conducted in Egypt [8], India [9, 10], and Iran [11] have attempted to develop socioeconomic or economic status measurement questionnaires and/or to improve existing ones for health research and welfare assessments. These attempts found the possibility of estimating the socioeconomic status of each household and individual using a limited number of indices. In Cambodia, poor households are identified using the proxy means test [12]. However, no study has so far focused on measuring the household income level applicable to health insurance contribution estimation.

Accordingly, the present study aims to develop and validate an efficient household income-level assessment model for Cambodia by using only selected independent variables and respective regression coefficients. The final product of the study will be an automated tool that predicts the income level of a household, which will further determine the optimal amount of health insurance contribution for each household. If the model determines that subnational administrative location is relevant for predicting income, it will be included in the tool, thus rendering the model useful for nationwide application. This will help the country implement a financially sustainable social health insurance system in which the insured can pay contributions according to their ability.

## Methods

### Data source

This study will use the CSES 2009–2019 data [7, 13–20], provided by the NIS, that is publicly accessible on request. These data contain demographic characteristics; housing conditions; and household-level production, income, consumption, and ownership of assets. The CSES is a nationally representative cluster sample survey using systematic sampling with probabilities proportional to the size of the stratum. The sample sizes for urban and rural areas are calculated using the proportion of consumption in the two parts of the population with the preceding CSES data. The interview was conducted for the head of the household, his/her spouse, or any other adult household member if the head and spouse were absent. The NIS conducts the survey annually, and it covered 12,000 households in 2009, 3600 in 2010–2013, 12,096 in 2014, and 3840 in 2015–2017. The CSES was not conducted in 2018 as the NIS was revising the questionnaires. Although the details regarding CSES 2019 have not been published, we expect to include the CSES 2019 data in our analyses based on the timeline for data availability from the NIS.

For this study, we plan to use data from the 50,016 households covered in the CSES 2009–2017, plus those from CSES 2019. We will use pooled cross-sectional data from several years to utilize a larger sample size that would increase precision and power, and allow assessment of the effect year in the estimation of income.

To define income composition and distribution for households, the CSES uses the Recommendations on Household Income Statistics by the Canberra Expert Group [7]. The CSES estimates household income by asking over 60 questions regarding revenue and costs of each economic activity, including those related to agriculture, non-agriculture, and owner-occupied houses, and other types of income and transfers. Since there is no bookkeeping in households, one has to rely on data from interviews on both revenue and costs for the households as business. The CSES’s estimation is based on the recall and diary data on the items listed in Table 1.

**Table 1:** Composition of household income

1. Primary income	
1.1	Salary
1.2	Agricultural income
1.3	Non-agricultural income
1.4	Income from owner’s house occupied
1.5	Income from property
2. Total transfers	
2.1	Total private transfers
2.2	Scholarship
2.3	Gifts
2.4	Other transfers
2.5	NGO transfers
2.6	Pension
3. Total income (1 + 2)	
4. Negative transfers	
4.1	Taxes
4.2	Inter-household transfers
4.3	Cash transfers to charities
5. Disposable income (3 - 4)	

Source: Cambodia Socio-Economic Survey 2014 [7]

### Analyses

The data will be divided into a training set and a validation set using the 9:1 ratio randomly. This training- and validation-set size ratio was determined to optimize the trade-off between the complexity of the first and second levels of inference [21]. Subsequently, the analyses will be conducted in two steps.

In the first step, using the training set, we will perform elastic net regression with per capita disposable income based on purchasing power parity as the dependent variable and the socioeconomic factors listed in Table 2 as independent variables. Here, we will use the variables discussed in similar studies to create our pool of independent variables. Household size could be an independent variable as it affects per capita household income, while it is also used for computing the average income of each household member. Then we will apply elastic net regression to identify variables that are most relevant for predicting income given the context [8–11]. Elastic net regression is a widely applied technique that performs data-driven automatic variable selection and regularization to optimize predictive performance on future data while ensuring model parsimony [22, 23]. Per capita disposable income in international dollars will be derived from per capita disposable monthly income in the local currency, using a purchasing power parity conversion factor from the World Bank [24]. This will ensure the international comparability and validity of the findings over time within the country, given the volatility of the local currency and prices. To account for the variance in the estimation of per capita disposable income based on purchasing power parity, the household size used to estimate per capita disposable income in each household will be used to construct analytic weights for the modelling framework. K-fold cross-validation will be used to select the optimal alpha and lambda hyperparameters for the model. Additionally, we will conduct subgroup analyses, which will allow us to develop separate income-level assessment models for each socioeconomic group of the society, including the urban and rural areas, administrative regions, and sectors of economic activity, taking their socioeconomic heterogeneity into account.

**Table 2:** List of variables tested in the development of the income assessment framework

Dependent variable	
• Disposable per capita income	
Independent variables	
Demographic characteristics	
• Household size	
• Whether there is no male/female in a household	
• Sex, age and ethnicity of the household head	
• Whether the household head has a spouse	
• Number of household members who received college-level or higher education	
• Number of household members who received high-school-level or higher education	
• Number of literate household members	
• Number of household members who currently go to private school	
• Number of household members who are currently receiving private lessons	
• Total payment for household members’ education in the past year	
• Number of household members who speak French/English/Chinese	
Geographic characteristics	
• Urban or Rural (Primary Sampling Unit basis) residence	
• Provincial/district/commune/village code	
Housing	
• Materials used for the dwelling roof/wall/floor	
• Source of light/drinking water	
• Type of toilet facility/cooking fuel	
• Whether/how the household treated drinking water in the past month	
• Water/sewage/garbage/electricity/gas bill	
Property	
• The number/price of the household’s buildings	
• Whether the household rents out buildings	
• Rent value of the household’s buildings	
• Whether the household renovated buildings	
• Number of households sharing the dwelling	
• Area size of the dwelling	
• Number of rooms in the dwelling	
• Rent that the household paid in the past month	
• Amount that the household paid for maintenance of the own buildings	
• Whether the household owns the dwelling	
• Whether the household owns or operates a pond	
• Number of ponds the household owns	
• Total area size/price/monthly rent value of the household’s ponds	
• Number of land plots that the household owns or operats	
• Total area size/total value of the household’s lands	
• Whether the household owned or operated land for farming in the past year	
• Number of wet-season/dry-season land plots that the household owns	
• Number of land plots that the household grows rice/non-rice crops/permanent crops	
• Whether the household can add water to land in the wet/dry season	
Liability	
• Whether the household has an outstanding loan	
• Number/total amount of the household’s outstanding loans	
• Whether the household sold land in the past year	
Livestock	
• Whether a household member raised livestock in the past year	
• Number/value of cattle/buffaloes/pigs/chicken/duck/ that the household has	
• Whether a household member raised/caught fish in the past year	
Economic Activities	
• Number of income-earners in the household	
• Total number of household members working in foreign countries	
• Whether a household member grew/stored/sold crops in the last season	
• Total crops that the household produced in the last season	
• Whether a household member ran a business or enterprise in the past year	
• Total household members employed by the government/enterprise/household sector/aid organization, etc.	
• Total household members who are employers/employees/self-employed, etc.	
• Total household members who are technicians/professionals/managers/service and sales workers/skilled agricultural workers/craft workers/plant operators/technicians, etc.	
Durable Goods	
• Whether the household has an AC/ bed/ bicycle/ bulldozer/ cabinet/ camera/ car/ cart/ cell phone/ computer/ dining set/ dish washer/ electric fan/ electronic iron/ freezer/ gas kitchen/ generator/ hand tractor/ harrow/ motorcycle/ motor boat/ music instrument/ phone/ plough/ printer/ pump/ radio/ refrigerator/ rice mill/ rowing boat/ satellite/ sewing machine/ sofa set/ sport good/ stereo/ threshing machine/ tractor/ TV/ vacuum/ video/washing machine	
• Number of the above durable goods that the household owns	

Source: Cambodia Socio-economic Survey 2009–2017 [7, 13–20]

In the second step, the trained model will be validated using the validation data subset. Using this subset, we will predict the income level of each household and further classify the households into income quintile groups. The results will also be compared with the income quintiles reported by the CSES, which used the full-length income-assessment questionnaires. The same data subset of the CSES 2009–2019 will be utilized using the F-score, which represents the harmonic mean of the positive predictive value and true positive rate [23]. By using large data, we have made the ratio of training- and validation- datasets larger than usual, at 9:1, as 5000 observations are large enough for validation. Finally, we will conduct sensitivity analyses to explore the effect of using different training- and validation-set size ratios on our inferences. All analyses will be conducted in Stata 15.0 and R 3.5.1. A *P* value < 0.05 will be considered statistically significant.

## Discussion

This research will help Cambodia implement a sustainable social health insurance system by collecting an optimal amount of contribution from each socioeconomic group of the society. For the first time, our approach will allow for an equitable contribution collection from all the households by determining a pool of a few highly predictive indices that can reliably provide an accurate estimation of per capita income, while ensuring feasibility of the insurance fund by allowing informed planning with accurate estimation of the revenue pool. Incorporation of our proposed insurance framework into the existing NSSF schemes will thus enhance the country’s current efforts to prevent impoverishing health expenditure and to achieve UHC targets. Our results will be compared with the fixed health insurance contribution estimated in a previous study based on medical benefit costs, administration costs, and capital buffer added, as illustrated in Table 3 [26]. Furthermore, applicability of the income-assessment tool will be tested in a subsequent pilot study.

**Table 3:** Projected fixed and equitable contributions per capita by quintile groups in Cambodia, 2019

			Fixed contribution		Equitable contribution	
	Projected income (USD)	Projected income distribution	Projected contribution (USD)	Projected contribution share in income	Projected contribution (USD)	Projected contribution share in income
I	19.0	2.6%	3.3	17.3%	0.4	2.2%
II	76.0	10.4%	3.3	4.3%	1.7	2.2%
III	116.8	16.0%	3.3	2.8%	2.6	2.2%
IV	166.9	22.8%	3.3	2.0%	3.8	2.2%
V	352.0	48.2%	3.3	0.9%	7.9	2.2%

Note: 1 USD = 4050.58 Cambodian Riels [25]. Quintile I and V are the lowest and highest income groups, respectively. Source: Japan International Cooperation Agency [26]

Despite our innovative methodology to estimate income, there are some practical difficulties that would need to be addressed in the field to collect these contributions. These include unwillingness to make payments, particularly for an unfamiliar expense such as health insurance. Even if individuals pay their contributions, embezzlement could be a problem. Additionally, it might be costly to collect contributions. Therefore, additional efforts from the Government of Cambodia might be required for the successful implementation of our approach. Actions could include legislating compulsory health insurance contributions, setting up a mechanism of peer pressure in the contribution collection, using electronic money transfer methods, including automatic withdrawal from bank account, as well as utilizing the existing local administrative system to collect contributions. Furthermore, though the present study will not use self-reported income as this will be predicted using non-income data, there is still the issue of social desirability and recall bias which constitute the limitations of the study.

## Data Availability

The datasets that support the findings of this study are available from the National Institute of Statistics (NIS), Ministry of Planning of Cambodia. Data are available upon reasonable request and with permission of the NIS.

## Abbreviations

CSES: Cambodia socio-economic survey
LMICs: Low- and middle-income countries
NIS: National institute of statistics
NSSF: National social security fund
UHC: Universal health coverage
